# Adaptive Capacities and Complexity of Heart Rate Variability in Patients With Chronic Obstructive Pulmonary Disease Throughout Pulmonary Rehabilitation

**DOI:** 10.3389/fphys.2021.669722

**Published:** 2021-07-28

**Authors:** Louis Hognon, Nelly Heraud, Alain Varray, Kjerstin Torre

**Affiliations:** ^1^EuroMov Digital Health in Motion, University of Montpellier, IMT Mines Ales, Montpellier, France; ^2^Direction de la Recherche et de l’Innovation en Santé – Korian, GCS CIPS, Lodève, France

**Keywords:** fractal, exercise capacities, 6-minute walking test, perturbation, chronic disease, resilience

## Abstract

**Introduction:**

The complexity of bio-signals, like R-R intervals, is considered a reflection of the organism’s capacity to adapt. However, this association still remains to be consolidated. We investigated whether the complexity of R-R intervals at rest and during perturbation [6-minute walking test (6MWT)], yielded information regarding adaptive capacities in Chronic Obstructive Pulmonary Disease (COPD) patients during pulmonary rehabilitation (PR).

**Methods:**

In total, 23 COPD patients (64 ± 8 years, with forced expiratory volume in 1 s of 55 ± 19% predicted) were tested three times at the start (T1), middle (T2), and end (T3) of 4 weeks PR. Each time, R-R intervals were measured at rest and during 6MWT. The complexity of R-R intervals was assessed by evenly spaced Detrended Fluctuations Analysis and evaluated by the fractal exponent α and deviation from maximal complexity |1-α|.

**Results:**

The 6MWT distance was significantly increased at T2 and T3 compared to T1. Neither α nor |1-α| at rest and during perturbation significantly changed throughout PR, nor were they consistently associated with 6MWT distances at each time. Throughout the PR program, complexity during the 6MWT was significantly lower compared to the rest. The level of α during 6MWT at T1 was positively correlated with the improvement of the 6MWT distance throughout the PR program.

**Discussion:**

Reduced complexity in COPD patients during acute perturbation at the beginning of PR supports a decreased improvement of the 6MWT distance throughout PR. This result seems consistent with the notion that the complexity reflects the patients’ adaptive capacities and could therefore become a clinical indicator in an applied perspective.

## Introduction

The systemic and not fully reversible aspects of chronic diseases, such as Chronic Obstructive Pulmonary Disease (COPD), and the difficulty in understanding their complex causalities have led to consider the health status in these chronic conditions as closely related to adaptive capacities (Heylighen, 1999; Sturmberg, 2014; Sturmberg et al., 2015; Malara, 2018). Adaptive capacities can be defined as the ability of an individual or a physiological system, such as the cardiorespiratory system, to manage internal or external perturbations *via* behavioral changes allowing for the organism’s homeokinesis, taking into account one’s intrinsic condition-induced limitations (Iberall and McCulloch, 1968; Goldberger and West, 1987; Amagoh, 2008; Macklem, 2008; Huber et al., 2011; Seely and Macklem, 2012). This ability results from interaction-dominant dynamics within the system, with its components being hardly assessable or treatable in isolation (Ahn et al., 2006; Fang and Casadevall, 2011). Mostly, interaction-dominance yields complex, fractal patterns of variability in the physiological signals generated by the system (Peng et al., 1995, 2009; Korolj et al., 2019). Thus, different methods were developed to evaluate and report properties of the fractal complexity of physiological signals (Heylighen, 1999; Harbourne and Stergiou, 2009; Bravi et al., 2011; Van Orden et al., 2011). The complexity measures of time series have been considered an indicator of adaptive capacity levels (Peng et al., 2009; Manor et al., 2010; Korolj et al., 2019), associated with the risk of adverse events or mortality in the case of decreased complexity levels (Bodduluri et al., 2018; Sen and McGill, 2018; Li et al., 2019a; Raichlen et al., 2019). However, the association between fractal complexity within physiological time series and adaptive capacities has not yet been established in a clear manner.

Indeed, this association is mainly an indirect one, based on cross-sectional studies showing a reduced complexity in groups of patients and in several medical conditions compared to healthy individuals (Vaillancourt and Newell, 2002; Carvalho et al., 2011; Manor and Lipsitz, 2013; Dames et al., 2014; Li et al., 2019b). Moreover, considering that adaptive capacity is defined as the capacity to manage perturbations, it is quite irrelevant for most studies to assess complexity in baseline situations without evaluating the system’s response to acute or chronic perturbations (Ideker et al., 2001). In a general perspective of using complexity as a clinical indicator of health status, this association clearly deserves to be strengthened, mainly through longitudinal approaches investigating the relationship between the complexity of time series and the evolution of the system’s responses under perturbation (Ideker et al., 2001; Gijzel et al., 2019). In the context of pulmonary rehabilitation (PR) in COPD, these advances would help bring answers to a dual clinical challenge. Firstly, besides the objective of improving patients’ functional capacities, PR is also meant to improve their adaptive capacities to better manage future events (Lacasse et al., 2007; Spruit et al., 2013). Therefore, it is necessary to have tools to measure patients’ adaptive capacities and to assess their evolution during PR. Secondly, one of the current challenges lies in predicting the patient’s response—or capacity to adapt favorably—to PR [the evolution of the 6-minute walking test (6MWT) distance throughout PR]. This underlying challenge is highly relevant since the 6MWT distance over time has been associated with increased mortality risks (Pinto-Plata et al., 2004; Cote et al., 2007; Polkey et al., 2013). Additionally, this could enable detection COPD patients with limited adaptive capacities.

Physical exercise is likely to highlight the association between the complexity of time series and adaptive capacities in COPD patients during PR. Indeed, the level of complexity of physiologic time series has previously been associated with chronic physical activity: physical activity programs improve the complexity of physiological signals at rest, especially cardiac variability (R-R intervals series) (Heffernan et al., 2007; Soares-Miranda et al., 2011; Millar et al., 2013). Contrarily, in a situation of acute physical perturbation (e.g., incremental cycling exercise), it was evidenced that the level of complexity of R-R intervals series was reduced along with the increasing intensity of the exercise, revealing the organism’s adaptive capacities to stress (Hautala et al., 2003; Casties et al., 2006; Gronwald et al., 2019b). In the specific context of COPD, a physical training program was shown to improve both exercise tolerance (6MWT and complexity of R-R interval series at rest (Borghi-Silva et al., 2015), which is congruent, without being conclusive, to the association between complexity and adaptive capacities since the complexity of R-R intervals assessment during perturbation was not assessed.

The objective of this study was to test the association between adaptive capacities and fractal complexity of physiologic time series in the context of COPD, *via* a longitudinal approach addressing the organism’s response to perturbation throughout PR. Chronic adaptation to PR and exercise tolerance in COPD are typically assessed by the 6MWT (Butland et al., 1982; Ben Cheikh Rejbi et al., 2010; Ambrosino and Clini, 2015). In addition, in the short term, the 6MWT represents a major perturbation for COPD patients, involving high exercise intensity and increased heart rate and dyspnea levels (Troosters et al., 2002; Vonbank et al., 2020). Considering the above, we hypothesized that the complexity of R-R intervals should improve during PR, in association with an improvement in 6MWT distance. In contrast, the complexity of the R-R interval series assessed during the 6MWT should decrease vs. resting condition. Finally, if the complexity of the physiologic time series reflects patients’ adaptive capacities, including their response to PR, the complexity level of R-R intervals at the beginning of the PR program should be associated with the improvement of the 6MWT distance throughout PR.

## Materials and Methods

### Study Participants

In total, 23 COPD patients were involved in a 4-week PR program. COPD diagnosis and classification of airflow obstruction severity followed the Global Initiative for Chronic Obstructive Lung Disease (GOLD) guidelines (Vogelmeier et al., 2017). Patients were classified as mild (GOLD I), moderate (GOLD II), severe (GOLD III), or very severe (GOLD IV). Non-inclusion criteria were: exacerbation within 4 weeks prior to the patient’s arrival at the clinic, a rehabilitation carried out within 1 year prior to inclusion, a cardiac device, any unstable cardiac condition (e.g., atrial fibrillation and arrhythmias), and neuromuscular or orthopedic diseases affecting gait. The test for cardiac conditions was administered by a medical doctor before the start of the PR program. It consisted of recording heart rate variability at rest and during a symptom-limited exercise tolerance test on a cycle ergometer. During the entire PR program, patients were taking their routine medical treatment, no changes or adjustments were made. All patients signed a written consent form prior to the study, and the study protocol was approved by an independent Ethics Committee (CPP: 2019-A005582-55).

### Study Design

The study was designed as a multicenter longitudinal study, involving the “La Solane,” “La Vallonie,” and “Les Clarines” “Cliniques du Souffle” PR centers in Osséja, Lodève, and Riom-ès-Montagnes (France). Pulmonary function and anthropometric variables were measured upon arrival at the clinic. Each patient performed a 4-week PR program. They participated in three experimental sessions, each separated by a 2-week interval and integrated into the PR program at the start: T1, middle: T2, and end: T3. Each session included two experimental tests: R-R intervals were recorded at rest and during 6MWT.

### Pulmonary Function Tests and Anthropometric Variables

The pulmonary function of COPD patients was tested using a plethysmograph (V6200 Autobox, Sensormedics Corp., Yorba Linda, CA, United States), assessing the following variables (Vogelmeier et al., 2017): forced vital capacity (FVC), forced expiratory volume at the first second of a maximal expiration (FEV_1_), Tiffeneau ratio (FEV_1_/FVC), and total lung capacity (TLC). Except for Tiffeneau ratio, which was expressed only in measured values, variables were expressed in measured values and as a percentage of predicted values. The following anthropometric variables were measured: height (cm), weight (kg), and body mass index (BMI) (kg.m^–2^).

### Pulmonary Rehabilitation Program

The PR program was carried out according to recommendations from the American Thoracic and European Respiratory Societies (Maltais et al., 2014). It was based on an exercise training program and educational therapeutic sessions prescribed based on a comprehensive medical check-up at the start of the PR program. The exercise training program included endurance training (average 7 h/week), resistance training both for upper and lower limbs (average 2 h/week), balance and postural exercises, and relaxation (average 4 h/week). Therapeutic education, initiation to self-management, and group sessions on behavioral change were also provided, including nutritional, psychological, and nursing support (average 3 h/week). The PR program was comparable for the three different centers, thanks to a common training for all rehabilitation professionals.

### Experimental Procedures

To avoid the circadian rhythm’s influence on heart rate variability, all experimental sessions (T1, T2, and T3) were performed between 9:00 a.m. and 12:00 p.m. for all COPD patients. Patients were asked to abstain from smoking, consuming alcohol, or any drink containing stimulants (caffeine, theine, and taurine) in the 6 h prior to the session. Each session was composed of two testing times: R-R intervals were recorded first at rest, and then during the 6MWT. Testing under resting the condition took place in a quiet room at a constant temperature of 22°C. Patients were fitted with a polar V800 belt and watch, positioned in a bed in a lateral recumbent position for a 15-min recording of R-R intervals. Patients were instructed not to move or speak, and to continue breathing normally during the recording. Immediately after the resting condition, patients were directed to a quiet 30-meter corridor for recording under the 6MWT condition. At the end of the 6MWT, the following parameters were recorded: distance walked, mean heart rate, and dyspnea level. Perceived fatigue was assessed at the beginning of each session before the resting phase.

#### R-R Intervals Acquisition

In order to assess the complexity of heart rate variability, we recorded R-R intervals by using a portable heart rate monitor, Polar V800, with a Polar H10 chest strap (Kempele, OY, Finland). The polar V800 system consists of a watch worn on the left wrist and connected by Bluetooth to an electrode belt fitted on the chest, which records R-R intervals at a sampling frequency of 1000 Hz. It was validated for recording R-R intervals under resting and exercise conditions (Giles et al., 2016; Caminal et al., 2018). Before the start of R-R intervals recording, the patient’s skin was cleaned and prepared for receiving the polar V800 electrode belt. Then, the belt was humidified and adjusted tightly, but comfortably, just below chest muscles in accordance with the device’s instruction. The raw R-R intervals were exported from the Polar Flow web service as a space delimited.txt file.

#### The 6-Minute Walking Test

The 6MWT was administered in accordance with the American Thoracic Society guidelines at three different times during the PR program (Spruit et al., 2013). Patients were instructed to walk as far as possible for 6 min. At T1, in accordance with good clinical practices and recommendations, two 6MWTs were performed for each patient, separated by a 30-minute interval. For the purpose of the experiment, and in order to ensure that heart rate variability analyses were carried out during the very first perturbation phase after the resting period, we elected to use the first 6MWT performed for analyses. The individual responses to PR were then calculated as the difference between 6MWT distances at T3 and T1.

#### Dyspnea Level

For quantifying the physiological and respiratory impact of 6MWT on COPD patients throughout PR, we collected the mean heart rate (beats.min^–1^) and dyspnea level during the 6MWT. Dyspnea level was measured before and after the 6MWT on a visual analog scale ranging from 0 to 10, with 0 representing no respiratory discomfort and 10 sensations of asphyxia.

#### Perceived Fatigue

Pulmonary rehabilitation-induced fatigue in COPD patients would likely cause alterations in the complexity of time series (Pethick et al., 2015, 2016). In order to control this putative confounding factor, we assessed perceived fatigue using a validated multidimensional questionnaire (MFI-20) (Smets et al., 1995; Gentile et al., 2003), at the very beginning of each experimental session, before conducting any testing.

### Data Analysis

#### R-R Intervals Pre-processing

The processing and artifact corrections in the recorded R-R intervals were carried out using the RHRV package on R software (Martínez et al., 2017). First, R-R interval series were visually inspected to detect a stable window for analysis of at least 400 R-R intervals, with few artifacts. In order to analyze RR intervals over stable periods of time, suitable for the assessment of complexity properties (Almurad and Delignières, 2016; Liddy and Haddad, 2018), the windows of R-R interval series were mainly selected at the end of the resting phase, and after the second minutes of the 6MWT. Indeed, during the first 2 min of 6MWT, COPD patients show a higher average speed (8), and various physiological parameters grow rapidly, such as the average heart rate and V̇O_2_ (Troosters et al., 2002; Vonbank et al., 2020). Such instability could impact the quality of the R-R intervals by causing numerous artifacts. However, after the second minute, COPD patients reach a plateau in V̇O_2_, and their average speed and heart rate tend to stabilize (Troosters et al., 2002). Therefore, from the second minute until the end of the test, the intensity of 6MWT test is relatively constant and allows for a proper assessment of R-R intervals with a portable device like the Polar V800. Putative artifacts were detected from these selected windows and removed if they were not 95% similar to the 10 previous ones and the 10 following ones. In cases where several artifacts were grouped together and not automatically detected, but visually identified, they were removed manually *via* the RHRV package. After artifact correction, a series of 256 R-R intervals was retained for each participant under each experimental condition for subsequent analyses, in accordance with previous guidelines (Shaffer and Ginsberg, 2017) and in order to have the same number of intervals for each subject (Ribeiro et al., 2018). Series of R-R intervals were linearly detrended before evaluating their complexity, in accordance with standard methodology (Peng et al., 1995; Delignières et al., 2005; Arsac and Deschodt-Arsac, 2018).

### Characterizing the Complexity of R-R Interval Series

The complexity of the R-R interval series was assessed by the fractal exponent α yielded by evenly spaced Detrended Fluctuation Analysis (DFA; Almurad and Delignières, 2016; Liddy and Haddad, 2018). DFA was used because it provides reliable results for “short” time series, especially for 256 data (Almurad and Delignières, 2016; Liddy and Haddad, 2018). The DFA methodology was previously described (Delignières et al., 2005; Almurad and Delignières, 2016; Arsac and Deschodt-Arsac, 2018; Liddy and Haddad, 2018). To sum up, DFA analyses the relationship between the average fluctuation amplitude and the size of the observation window where these fluctuations are measured. First, the R-R interval series is integrated, then it is divided into *N*_*n*_ adjacent segments of length *n* (with *n* ranging from 4 to 64). Within each segment of the integrated series, the linear trend is locally removed. Secondly, the characteristic size of the fluctuation *F*(*n*) is calculated as the root mean square deviation from the trend for each segment size. If the time series is self-similar, or fractal, a power-relationship characterized by the fractal exponent α € [0, 2] is expected: *F*(*n*) *n*^α^. The exponent α is estimated as the slope of the linear regression fit to the log-log graph of *F*(*n*) against *n*.

Nevertheless, log–log coordinates cause an overweighting of the large time windows (*n*) in the plot, which is likely to skew the exponent α estimation. Therefore, recent methodological developments showed that evenly spacing (i.e., geometrically equidistant points instead of logarithmically spaced points in the plot) improves the reliability of α estimates (Almurad and Delignières, 2016; Liddy and Haddad, 2018). We included this procedure in our present analysis, prior to α estimation. As a result of DFA, for α = 1 the complexity of the time series is maximal, indicating strong coordination among the components of the system, providing it with adaptive capacities. For α tending toward 0.5, the series is Gaussian white noise (totally random time series), suggesting a loss of coordination among the systems’ components. For α tending toward 1.5, the series is non-stationary, Brownian motion, suggesting a very rigid and tightly controlled system. Both deviations from 1 represent a lower complexity of the series, not allowing the system to show a globally efficient adaptive response to perturbation (Kello et al., 2010; Diniz et al., 2011; Van Orden et al., 2011; Richardson et al., 2014). In order to express the deviation of the fractal exponent from maximal complexity (whether >1 or <1), we calculated the deviation from the maximal complexity index as |1-α|. DFA was implemented in Matlab R2015a software.

#### Analyses of R-R Interval Series in Temporal and Frequency Domains

In order to ensure the consistency of R-R interval data at baseline and during the PR program, we performed supplementary temporal and frequency analyses following the 1996 task force recommendations (Electrophysiology Task Force of the European Society of Cardiology the North American Society of Pacing, 1996).

The assessment of the R-R interval in the time domain was executed with SDNN (standard deviation of normal-to-normal N-N intervals; R-R intervals after artifact corrections) and RMSSD (root mean square of R-R intervals). In the frequency domain, we determined the low frequency (LF: 0.04–0.15 Hz) spectral component and the high frequency (HF: 0.15–0.40 Hz) spectral component. HF and LF powers were normalized in regards to the total variance in order to express relative contributions of spectral components, with LFnu = LF/(LF + HF) and HFnu = HF/(LF + HF). Afterward, we assessed the LF/HF ratio between both components, i.e., the ratio between sympathetic and parasympathetic activity (Shaffer and Ginsberg, 2017). During the 6MWT the upper limit of the HF band was increased to 1 Hz to ensure that breathing frequency during exercise was taken into account (Malliani, 1999). The very low frequency band (VLF) was not measured due to its known limitations in the short-term analysis (Electrophysiology Task Force of the European Society of Cardiology the North American Society of Pacing, 1996). For these analyses, we used the RHRV packages in R studio software.

#### Statistical Analysis

Values are represented by the mean ± standard deviation in the case of normal distribution (tested with Shapiro–Wilk test), otherwise by the median [lower quartile–upper quartile]. The evolution of the fractal exponents α and |1-α| as well as the temporal and frequency domains of R-R interval series at rest and during the 6MWT, over the three different times of the PR program, was assessed using a two-way repeated-measures ANOVA 3 (time) × 2 (condition). Variables stemming from the 6MWT (distance, mean heart rate, and dyspnea), MFI-20 scores were compared over the three PR program times using a repeated-measures ANOVA (three times). The assumptions of sphericity were tested using Mauchly’s test. In case of violation of the assumption of sphericity, type 1 risk was corrected by the Greenhouse–Geisser coefficient. In case of violation of normality, we used the non-parametric Friedman test. If analyses showed a significant difference between the three times, we carried out *post hoc* analyses with Tukey adjustment or with Conover test in a non-parametric situation. We used Pearson’s correlation tests to study, on the one hand, the relationship between fractal exponents and 6MWT distances at each time, and, on the other hand, the relationship between fractal exponents at T1 and the improvement of 6MWT distance over PR. The significance level was set at *p* ≤ 0.05. All statistical tests were carried out with R and R studio software.

## Results

### COPD Patient Characteristics

For the 23 patients included in the study, 1 was GOLD stage I, 12 were GOLD stage II, 7 were GOLD stage III, and 3 were GOLD stage IV (Table 1).

**TABLE 1:** Demographic, anthropometric, and pulmonary function data of COPD patients.

**Demographic/anthropometric**	
F/M (*n*)	10/13
Age (years)	64.3 ± 8.4
Weight (kg)	79.5 ± 18.9
Height (cm)	165 ± 7.6
Body mass index (kg.m^–2^)	27.5 ± 5.5
**Pulmonary Function**	
FEV_1_ (L)	1.5 ± 0.51
FEV_1_ (% pred)	55.5 ± 19.1
FEV_1_/FVC (%)	52.8 ± 10.5
TLC (L)	6.9 ± 0.9
TLC (% pred)	118.5 ± 21.0
GOLD stage I *n* (%)	1 (4%)
GOLD stage II *n* (%)	12 (52%)
GOLD stage III *n* (%)	7 (31%)
GOLD stage IV *n* (%)	3 (13%)

*FEV_1_, forced expiratory volume in one second; FVC, forced vital capacity; TLC, total lung capacity; GOLD, global initiative for chronic obstructive lung disease.*

### Response of COPD Patients to Pulmonary Rehabilitation

#### Evolution of the 6MWT Distance, Mean Heart Rate, Dyspnea Level, and Perceived Fatigue Throughout PR

**FIGURE 1:**
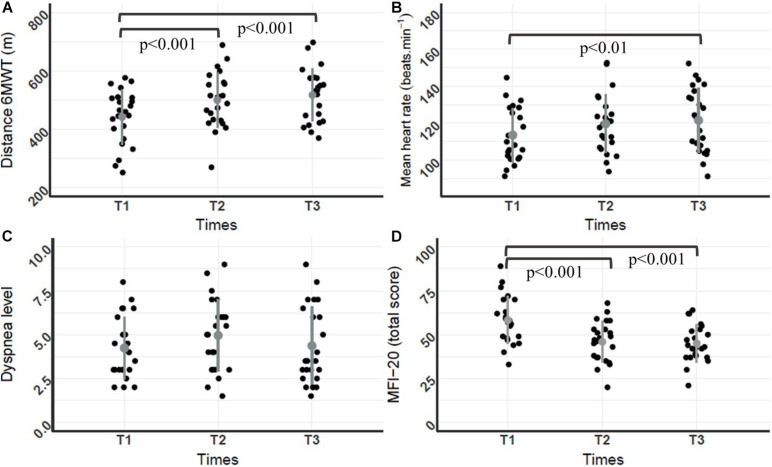
Dot plot of individual distances, mean heart rates, dyspnea levels during the 6MWT, and MFI20 scores throughout the PR program. **(A)** 6MWT distance (m); **(B)** mean heart rate (beats.min^–1^); **(C)** dyspnea level; **(D)** MFI-20 score; (*n* = 23 for **A–D**). Mean and standard deviation are represented by a dot and a line both in gray. Repeated ANOVA revealed a significant difference for the 6MWT distance, mean heart rate and perceived fatigue throughout the PR program [*F*(2,44) = 38.2, *p* < 0.05, η^2^ = 0.11; *F*(2,44) = 5.11, *p* < 0.05, η^2^ = 0.2; and F (2,44) = 23.74, *p* < 0.001, η^2^ = 0.21, respectively]. *Post hoc* analyses revealed that 6MWT distances at T3 and T2 were significantly higher compared to T1 (*p* < 0.001), mean heart rate during the 6MWT at T3 was significantly higher compared to T1 (*p* < 0.001) and MFI-20 scores at T3 (45.04 ± 10.5) and T2 (45.9 ± 11.7) were significantly lower compared to T1 (58.3 ± 13.6, *p* < 0.001).

The 6MWT distance, mean heart rate and perceived fatigue were significantly different throughout PR [*F*(2,44) = 38.2, *p* < 0.05, η^2^ = 0.11; *F*(2,44) = 5.11, *p* < 0.05, η^2^ = 0.2; and *F*(2,44) = 23.74, *p* < 0.001, η^2^ = 0.21], respectively}. *Post hoc* analyses revealed that 6MWT distances at T3 and T2 were significant higher compared to T1 (*p* < 0.001), and mean heart rate during 6MWT at T3 was significantly higher compared to T1 (*p* < 0.01) (Figure 1). Lowest scores to MFI-20 questionnaire corresponded to the lowest fatigue levels, and *post hoc* analyses revealed that scores at T3 (45.04 ± 10.5) and T2 (45.9 ± 11.7) were significantly lower in comparison to T1 (58.3 ± 13.6, *p* < 0.001) (Figure 1). No significant change was found for the dyspnea level.

#### Effect of Pulmonary Rehabilitation on the R-R Interval Series Analyses in Temporal and Frequency Domains at Rest and During the 6MWT

Only LFnu was significantly impacted by time and condition factors [*F*(2,42) = 3.44, *p* = 0.035, η^2^ = 0.036)]. Paired comparisons revealed that in resting condition, the LFnu score at T2 was significantly higher compared to T1 (*p* < 0.008), and during the 6MWT the score at T1 was significantly higher compared to the score T3 (*p* = 0.014). Concerning the others parameters, regarding the condition factor, both SDNN, RMSSD and HFnu were systematically higher in the resting condition than under 6MWT condition [*F*(1,21) = 37.2, *p* = 1.1e^–8^, η^2^ = 0.02; *F*(1,21) = 17.6, *p* = 4.9e^–05^, η^2^ = 0.01; and *F*(1,21) = 59.7, *p* = 2.49e^–12^, η^2^ = 0.01, respectively]. Conversely, LFHF was systematically higher in the 6MWT condition than under resting condition [*F*(1,21) = 90.2, *p* = 2e^–16^, η^2^ = 0.02] (Table 2).

**TABLE 2:** Effect of pulmonary rehabilitation on the analyses of R-R interval series in temporal and frequency domains at rest and during the 6MWT.

	Rest			6MWT		
		
	T1	T2	T3	T1	T2	T3
**Time-domain analysis**						
SDNN (ms)	18.6* (15.7–24.4)	23.5* (17.9–30.1)	23.0* (18.2–26.6)	7.8* (5.4–9.1)	8.1* (6.9–9.7)	7.1* (5.4–11.2)
RMSSD (ms)	12.9* (10.2–15.9)	11.5* (9.1–14.9)	11.8* (9.4–15.9)	4.5* (4.2–5.7)	4.4* (3.7–5.7)	4.52* (3.4–5.6)
**Frequency domain analysis**						
LF (nu)	59.2 ± 21.9*^#^	68.2 ± 13.5*^#^	65.9 ± 21.9*	33.6 ± 21.5*^‡^	33.0 ± 22.0*	29.3 ± 19.0*^‡^
HF (nu)	40.9 ± 21.9*	31.8 ± 13.5*	33.1 ± 21.9*	62.5 ± 21.5*	66.9 ± 22.0*	70.7 ± 19.1*
LF/HF	1.4* (0.8–3.5)	2.0* (1.4–4.1)	2.3* (1.1–4.3)	0.5* (0.2–1.2)	0.3* (0.2–0.7)	0.4* (0.2–0.9)

SDNN, standard deviation of NN intervals; RMSSD, root mean square of successive R-R intervals differences; LF (nu), low frequency in normalized units; HF (nu), high frequency in normalized units; LF/HF, low-frequency/high-frequency ratio.

*Significant difference between resting and 6MWT conditions at one time.

*^#^Significant difference for resting condition between times T1 and T2.*

*^‡^Significant difference for 6MWT condition between times T1 and T3.*

### Evolution Throughout the PR Program of the Complexity of R-R Intervals at Rest and During the 6MWT

**FIGURE 2:**
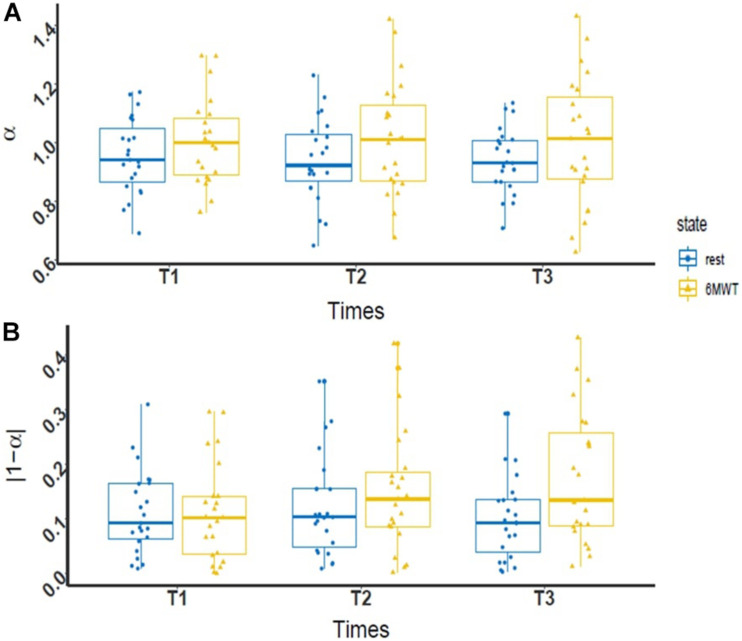
Box plot of R-R interval fractal exponent values during PR. **(A)** Evolution of the level of complexity (α) at rest (blue dot) and during 6MWT (yellow triangle) throughout the PR program (*n* = 23); **(B)** evolution of the deviation from maximal complexity (|1-α|) at rest (blue dot) and during the 6MWT (yellow triangle) throughout the PR program (*n* = 23). In each boxplot, the data are represented from bottom to top by the minimum value, the first quartile, the median, the third quartile, and the maximum value. A two-way ANOVA revealed a significant difference between complexity at rest and during the 6MWT for the two fractal exponents [*F*(1,21) = 0.09, *p* = 0.91, η^2^ = 0.001 and *F*(1,21) = 2.51, *p* = 0.09, η^2^ = 0.03, respectively].

For α and |1-α|, there was no significant interaction between time and condition factors [*F*(2,42) = 0.09, *p* = 0.91, η^2^ = 0.001 and *F*(2;42) = 2.51, *p* = 0.09, η^2^ = 0.03, respectively] (Figure 2). However, regarding the main factors, both α and |1-α| were systematically higher in the 6MWT condition than under resting condition [*F*(1,21) = 5.28, *p* = 0.045, η^2^ = 0.04 and *F*(1,21) = 4,81, *p* = 0.03, η^2^ = 0.03].

### Correlation Between R-R Interval Fractal Exponents and the 6MWT Distance at T1, T2, and T3

Amidst all possible correlations between the 6MWT distance and the measures of complexity α and |1-α| of R-R intervals at each time over PR, results revealed a significant correlation solely between the deviation from maximal complexity at rest and the 6MWT distance at T2 (*r* = −0.52, *p* = 0.01) (Table 3).

**TABLE 3:** Linear correlation coefficients (*r*) between fractal exponents at rest and during the 6MWT and 6MWT distance, throughout the pulmonary rehabilitation program.

	Fractal exponents	6MWT distance	*p*-value
**T1**	αrest	0.29	0.18
	α6MWT	–0.17	0.43
	|1-α|_*rest*_	–0.09	0.70
	|1-α| 6MWT	0.045	0.08
**T2**	αrest	0.26	0.24
	α6MWT	0.17	0.45
	|1-α|_*rest*_	–0.52	0.01*
	|1-α| 6MWT	–0.26	0.23
**T3**	αrest	0.20	0.37
	α6MWT	0.21	0.33
	|1-α|_*rest*_	0.05	0.82
	|1-α| 6MWT	0.14	0.54

*Significant association between fractal exponent and 6MWT distance (*p* < 0.05).

### Correlations Between R-R Interval Fractal Exponents at T1 and Patient Response to PR

**FIGURE 3:**
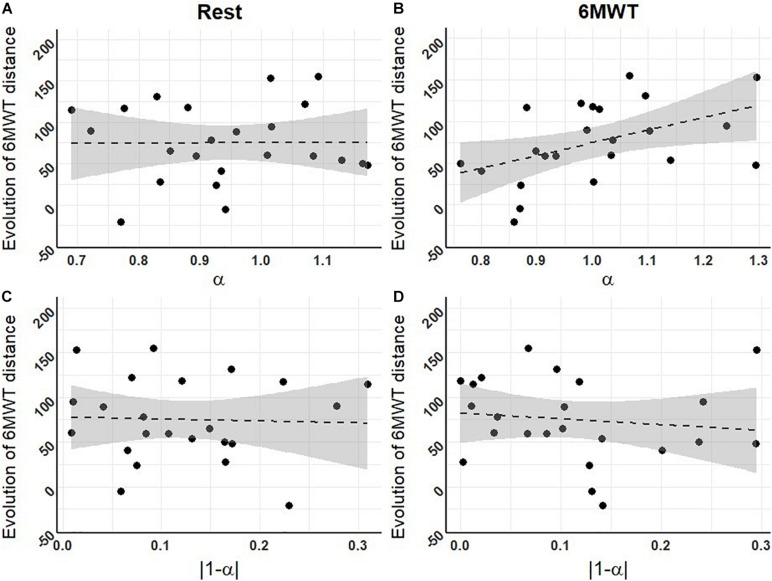
Correlations between fractal exponents at T1 and the evolution of 6MWT distances throughout the PR program. **(A)** Level of complexity (α) at rest; **(B)** level of complexity during the 6MWT; **(C)** deviation from maximal complexity (|1-α|) at rest; **(D)** deviation from maximal complexity during the 6MWT (*n* = 23 for **A–D**). Dashed lines represent the regression lines, with confidence intervals in gray. Pearson correlation tests revealed one significant positive correlation between the exponent α of the R-R interval series during the 6MWT at T1 and the improvement in 6MWT distance over PR (*r* = 0.47, *p* < 0.05).

Our results showed a significant positive correlation between the exponent α of the R-R interval series during the 6MWT at T1 and the improvement of 6MWT distance after PR (distance performed at T3 minus distance performed at T1; *r* = 0.47, *p* < 0.05, Figure 3).

## Discussion

The aim of this study was to put to the test the association between adaptive capacities and the complexity of physiological time series through a longitudinal approach in COPD, assessing the organism’s responses to acute perturbation (6MWT), and to PR. We found that patients’ response to PR was favorable, with an increase in the 6MWT distance and a decrease in perceived fatigue over time. In regards to complexity, we found no significant enhancement of the complexity level of R-R interval variability throughout PR, nor any consistent association of complexity (whether under resting conditions or during the 6MWT) with the 6MWT distance at the different PR program times. In contrast, when all times were considered together, the complexity was significantly lower during the 6MWT compared to the resting condition, and the level of complexity during the 6MWT at the start of the PR program was positively correlated with the increase of the 6MWT distance throughout the PR program.

### The Level of Complexity Does Not Reflect the Level of Functional Capacities Throughout PR

In our study, COPD patients increased their functional capacities (6MWT distance) throughout the PR program, but without showing any concomitant evolution of the structure of the R-R intervals’ fluctuations. This lack of change in the complexity of time series throughout PR may at a first appear inconsistent with previous results reported by Borghi-Silva et al. (2015). They investigated heart rate variability at rest and observed an improvement in the complexity of R-R intervals in COPD patients throughout a 6-week physical training program. Likewise, we did not observe any significant change of R-R intervals in RMSSD and SDNN during PR, in contrast to studies by Borghi-Silva et al. (2015) or Zupanic et al. (2014), in spite of similar training intensities. However, one should note that patients involved in these two studies presented with more severe airflow obstruction (mainly GOLD III and IV stages), and walked a lesser distance on the 6MWT both at the beginning and at the end of the PR program, compared to patients in the present study. It was established in COPD patients that a short walking distance is one of the main indicators of mortality (Pinto-Plata et al., 2004; Cote et al., 2007; Celli, 2010; Polkey et al., 2013) and therefore participates to the patient’s frailty status (Marengoni et al., 2018). At the same time, it was also shown that the perturbation effect, whether acute or chronic, on the variability properties of the physiological times series is stronger in frail individuals, due to an increased interdependence between the different components of the organism (Scheffer et al., 2009). Therefore, one can assume that including more frail patients in the present study would have likely shown an increased alteration of the properties for the R-R interval variability at the beginning of the PR program and that PR could have a greater impact on them. Nevertheless, frail COPD patients with several comorbidities (Vanfleteren et al., 2013) could present non-inclusion criteria considered in our study, especially arrhythmia (Müllerova et al., 2013). Thus, including patients with arrhythmia could have induced a potential confounding factor, since arrhythmia *per se* is known to decrease the complexity of R-R intervals (Glass et al., 1987). These elements point to a potential methodological dilemma when seeking to highlight a relationship between the complexity of R-R intervals and functional capacities of COPD patients during PR. Taking into account this methodological aspect along with the results of the present study underlines the notion that complexity level is unlikely to be a reliable marker of the evolution of functional capacities in COPD.

Consistently with the lack of change throughout PR of the complexity of time series and the hypothesis of independence between complexity and functional capacities, we found no correlation between the level of complexity and the 6MWT distance measured at the different times of the PR program. While this result differs from studies promoting an association between complexity and functional capacities (Cavanaugh et al., 2005, 2006; Stergiou and Decker, 2011; Manor and Lipsitz, 2013), it may trigger further discussions. Indeed, the literature hypothesized the existence of a threshold in pathological conditions: functional capacities may decline while preserving one’s capacity to adapt to imposed constraints until a threshold is reached where the level of constraints can no longer be managed by the organism given its functional capacities (Lipsitz, 2002). Therefore, one can assume that the level of complexity may be independent of functional capacities under different circumstances, for example, above a given threshold. In the present study, patients could hardly be considered below this threshold in light of their improved functional capacities during the PR program. As such, our results are not merely in contradiction with previous findings, but rather they emphasize the interest of considering the complexity of physiological signals in relation to adaptive capacities and apart from functional capacities.

### Only Complexity Measured Under Perturbation Relates to the Potential of Functional Improvement Throughout PR

In order to test the association between complexity and adaptive capacities, we assessed complexity under an acute perturbation (i.e., the 6MWT). In light of the previous results from the literature regarding the effect of acute physical perturbation on the level of complexity (Casties et al., 2006; Mazzuco et al., 2015; Gronwald et al., 2019b; Yentes et al., 2020), we expected to find a decreased complexity during the 6MWT compared to the resting condition. Moreover, given the assumed association between complexity and adaptive capacities, we expected that the complexity at the beginning of the PR program would relate to the patient’s response to PR. Our results are aligned with these two hypotheses.

We found a decreased complexity during the 6MWT compared to the resting condition, since the deviation from maximal complexity (|1-α|, Figure 2) was significantly higher during the 6MWT. This result matches the ones from the literature. Indeed, acute dynamic endurance exercise was previously reported to cause a loss of complexity in R-R intervals in healthy subjects (Hautala et al., 2003; Gronwald et al., 2019a,b). The 6MWT constitutes significant physiological stress for COPD patients (Troosters et al., 2002; Moreira et al., 2014; Vonbank et al., 2020). In the present study, it also impacted patients with an average four-point increase in dyspnea levels on the visual analog scale, and a mean heart rate increase. Interestingly, in our study, the level of complexity during the 6MWT was decreased, with α deviating from 1 toward 1.5 (Figure 2), instead of tending toward 0.5 as previously reported in the literature (Hautala et al., 2003; Casties et al., 2006; Gronwald et al., 2019a,b, 2020). This variation may be explained by specific characteristics of the 6MWT. Indeed, from the second minute of the 6MWT, the heart rate tends to stabilize (Troosters et al., 2002; Corrêa et al., 2013), thus yielding a physiological “steady” state during the course of the physical exercise, which is suitable for analyses. However, this state of equilibrium remains quite relative given the physiological stress imposed on patients during the 6MWT, triggering non-stationarities phenomena in time series, most likely leading to a global overestimation of the exponent α, tending toward 1.5 (Chen et al., 2002). In view of these elements, in our study the decreased complexity during the 6MWT compared to the resting condition seems consistent with the literature. It highlights the added value of assessing complexity both under resting conditions and under perturbation when looking at its relationship with adaptive capacities.

On the basis of the consistent difference between complexity during the 6MWT vs. resting condition, our results moreover show that a decreased exponent α during the 6MWT at the beginning of the PR program was associated with lower functional improvement (6MWT distance) in response to PR (Figure 3). This main result is consistent with the literature showing that a decreased complexity in several physiological signals and clinical conditions is associated with different poor outcomes, such as decreased cognitive function (Yang et al., 2013), increased risk of falls (Zhou et al., 2017), bad outcome after hospitalization (Tang et al., 2015), development of Alzheimer’s disease (Li et al., 2019b), and more generally an increased mortality risk (Mäkikallio et al., 1999; Batchinsky et al., 2007; Li et al., 2019a; Raichlen et al., 2019). The fact that the association between complexity and response to PR was only significant when complexity was assessed during perturbation (6MWT) is indeed consistent with previous results reported by Zhou et al. (2017): this study addressed the risk of falls in the elderly and showed that only the complexity of postural sway under dual-task perturbation was associated with the risk of falls in the upcoming months. Overall, this result could mean that unlike physiological complexity measures at rest, the way complexity is affected by a perturbation reflects the organism’s adaptive capacities and responsiveness.

### Complexity and Adaptive Capacities: Overview and Perspectives

To date, very few studies studied the association between complexity and adaptive capacities (Cavanaugh et al., 2006; Manor et al., 2010; Zhou et al., 2017; Ducharme et al., 2019). It was implicitly acknowledged, despite obvious limitations such as the absence of perturbation and longitudinal assessments in experimental designs. Accordingly, we believe that our study makes a step forward in characterizing the association between complexity and adaptive capacities. Additionally, it underlines the essential need for knowledge improvement in this new scientific field (Huber et al., 2011), especially concerning experimental means to assess adaptive capacities, since no methodological guidelines are available (Gijzel et al., 2019). Finally, our current results open both theoretical and applied perspectives.

First, on the theoretical side, adaptive capacities might be understood as a composite notion. Indeed, previous studies proposed to consider different dimensions of adaptive capacities (Conrad, 1977; Whitacre, 2010). In the short term, adaptive capacities may rely on robustness, i.e., the ability of a system to maintain itself within a limited functional range allowing it to operate as optimal (Kitano, 2007; Whitacre, 2010). In the long term, adaptive capacities may rely on the system’s ability to evolve, i.e., the ability to generate and select new/alternative behaviors to respond to constraints (Whitacre, 2010). In our present study, one can observe the two facets of the organism’s adaptive capacities. Indeed, comparing the complexity evaluated under resting condition vs. perturbation (6MWT) may reflect robustness, while complexity assessed under perturbation at the start of the PR program and correlated with the evolution of functional capacities throughout the PR program may be a marker of the capacity to evolve.

Second, on the applied side, the assessment of adaptive capacities may prove useful in the management of COPD patients, since identifying predictors for PR response is currently a major challenge. Response to PR itself constitutes a prognostic indicator for the ability to manage future events since a reduction in the 6MWT distance of 30 m or more is associated with a risk of death increased by twofold (Polkey et al., 2013), and 92% of patients walking less than 100 m die within 1 year post PR (Pinto-Plata et al., 2004). Knowing that one-third of COPD patients do not improve their 6MWT distance at the end of the PR program (Troosters et al., 2001; Spruit et al., 2015), the problem of response to rehabilitation is of crucial importance. The association found in this study between the initial complexity assessment and response to PR may yield significant clinical indicators to assess the adaptive capacities of patients.

### Physiological and Methodological Considerations

Our present study investigates the relationship between fractal properties of heart rate variability and adaptive capacities in COPD patients, taking a complex systems approach in the sense that the primary focus is on apprehending the complex dynamic interaction accounting for the complexity of heart rate variability and not on the identification of causal chains of mechanisms involved in the autonomic nervous system’s regulations. Nonetheless, some words may be needed to draw connections between these two complementary research paths. The mechanisms involved in cardiovascular regulation are non-linear (Goldberger, 1991; Goldberger et al., 2002), and DFA has been considered an adequate method to characterize its functioning, in particular in that it is known to partly represent the autonomic nervous system’s modulations (Perkiömäki et al., 2005; Gronwald et al., 2020). In this view, the α exponents close to 1 found in our present results in the resting condition could be interpreted as indicative of the integrity of the autonomic nervous system and its interactions with other physiological systems in patients (Peng et al., 1995; Gronwald et al., 2020). Conversely, according to Gronwald et al. (2019b), the increase of α toward 1.5 during 6MWT would indicate the reach of an equilibrium state between contributions of the sympathetic and parasympathetic systems, which leaves the cardiomyocytes of the sinus node cause a more strongly correlated or highly predictable heart rate (Gronwald et al., 2019b). However, seeing that our findings show a predominance of the high frequencies in heart rate variability during 6MWT, presumably resulting from the increase in arrhythmia of the respiratory sinus (Casadei et al., 1995), this hypothesis appears questionable. In parallel, the fact that our results show no significant changes in α exponents and frequency parameters of heart rate variability over the 4-week rehabilitation may indicate a lack of change in the autonomic nervous system, likely due to a duration of rehabilitation that is insufficient in this respect (Borghi-Silva et al., 2015). From a broader perspective, these considerations draw attention to the fact that spectral analyses in specific frequency bands and more global assessments of the complexity of heart rate variability come with their own specificities and limits in the condition of application and possible interpretations. For example, DFA offers more powerful prognostic information compared to frequency analyses (Perkiömäki et al., 2005; Sen and McGill, 2018), while frequency band analyses allow for deeper insight into the respective effects of the sympatic and parasympathetic system (Electrophysiology Task Force of the European Society of Cardiology the North American Society of Pacing, 1996). The specific information yielded by the two approaches thus deserves to be considered in a complementary way.

With more general regard to methodological choices, it might be worth noticing that non-linear approaches to heart rate variability, including DFA, provide a systemic assessment of the cardiac function as a result of the interaction between the autonomic nervous system and different electro-physiological variables, hemodynamic variables, etc. (Goldberger et al., 2002; Gronwald et al., 2020). That said, several factors such as comorbidities or drug treatments may be theoretically suspected to influence heart variability in COPD patients (van Gestel and Steier, 2010; Castiglioni et al., 2011), and they were taken into consideration in patients’ inclusion procedure. Nevertheless, patients with stabilized cardiac treatments or comorbidities were included in our present study, because selecting only patients without any drug treatment or comorbidity would be equivalent to studying a totally non-representative sample of COPD patients (Vanfleteren et al., 2013). Purposively designed mechanistic studies would therefore be suitable to determine the mechanisms influencing the properties of cardiac variability in COPD patients.

Finally, besides DFA several other non-linear methods exist to study cardiac variability. In our present study, the choice of DFA was primarily theory-driven, considering that different families of methods characterize different aspects or distinctive properties of complex dynamical systems (Bravi et al., 2011; Porta et al., 2017). For instance, some are considered better suited to characterize irregularity, such as entropy methods, others are chaos- or predictability-based markers, such as the Lyapunov exponent, and others are suited to characterize scale invariance, such as fractal methods. Within this landscape, and belonging to the fractal framework, DFA allows to apprehend the variability generated by a complex system in relation with a maximum complexity—or scale invariance in the strict sense (α close to 1) —considered as an optimal compromise between a totally disorganized (α close to 0.5) and a very tightly controlled (α close to 1.5) system (Van Orden et al., 2011; Almurad and Delignières, 2016). Now, our results may question the precise significance of the fractal properties of complexity, since we found that only α (and not |1-α|) measured during the 6MWT at T1 correlated with the improvement of 6MWT distance throughout rehabilitation. Considered in isolation, this result may suggest that it would be more the (ir)regularity of time series variability than the level of complexity with respect to scale invariance which may be significant in terms of adaptive capacities. Actually, however, it emphasizes once more the question of the conditions of congruence and the level of redundancy vs. specificity of the information provided by different families of methods developed to assess time series complexity. Indeed, one may for example consider that the fractal exponent of a time series to some extent also quantifies irregularity, besides scale invariance or fractal properties (Marmelat et al., 2012). It thus appears crucial to set up further studies dedicated to clarify the link between different non-linear methods and markers of complexity, in order to determine how they come together, and to better understand the specific significance of irregularity, predictability, chaos, and scale invariance, in terms of adaptive capacities.

## Conclusion

The current study shows that the complexity of R-R intervals is unlikely to reflect the level of functional capacities in COPD, seeing that fractal properties did not change throughout efficient physical rehabilitation, and were not associated with 6MWT distances. In contrast, it reveals that the fractal exponent during an acute perturbation at the beginning of PR is associated with the evolution of the 6MWT distance throughout rehabilitation. This association is consistent with the notion that fractal properties of complexity reflect adaptive capacities, and may become a clinical indicator in an applied perspective. Further studies should examine more precisely the relationship between the properties of the complexity of physiologic time series and adaptive capacities, with complementary experimental designs, methods, and the inclusion physiological perturbations.

## Disclosure

The results of this study are presented clearly, honestly, and without fabrication, falsification, or inappropriate data manipulation.

## Data Availability Statement

The raw data supporting the conclusions of this article will be made available by the authors, without undue reservation.

## Ethics Statement

The studies involving human participants were reviewed and approved by the Comité de Protection des Personnes, CPP: 2019-A005582-55. The patients/participants provided their written informed consent to participate in this study.

## Author Contributions

LH, NH, and KT conceived and designed the research. LH performed the experiments and prepared the figures. LH and AV analyzed the data. LH, NH, KT, and AV interpreted the results of experiments. LH and KT drafted the manuscript. NH, KT, and AV edited and revised the manuscript.

## Conflict of Interest

The authors declare that the research was conducted in the absence of any commercial or financial relationships that could be construed as a potential conflict of interest.

## Publisher’s Note

All claims expressed in this article are solely those of the authors and do not necessarily represent those of their affiliated organizations, or those of the publisher, the editors and the reviewers. Any product that may be evaluated in this article, or claim that may be made by its manufacturer, is not guaranteed or endorsed by the publisher.
